# Remarkable stability of $\gamma$-$N_2$ and its prevalence in the nitrogen phase diagram

**DOI:** 10.1038/s41598-024-66493-0

**Published:** 2024-07-16

**Authors:** Jinwei Yan, Philip Dalladay-Simpson, Lewis J. Conway, Federico Gorelli, Chris Pickard, Xiao-Di Liu, Eugene Gregoryanz

**Affiliations:** 1 Key Laboratory of Materials Physics, Institute of Solid State Physics, HFIPS, Chinese Academy of Sciences, Hefei, 230031 China; 2 Center for High Pressure Science and Technology Advanced Research, Shanghai, China; 3 Centre for Science at Extreme Conditions and School of Physics an Astronomy, University of Edinburgh, Edinburgh, UK; 4 University of Science and Technology of China, Hefei, China; 5 Department of Materials Science and Metallurgy, University of Cambridge, 27 Charles Babbage Road, Cambridge, CB30FS UK; 6 Advanced Institute for Materials Research, Tohoku University, Sendai, 980-8577 Japan; 7 Consiglio Nazionale delle Ricerche, Istituto Nazionale di Ottica, CNR-INO, Via Nello Carrara 1, 50019 Sesto Fiorentino (FI), Italy

**Keywords:** Condensed-matter physics, Structure of solids and liquids, Materials science, Physics, Condensed-matter physics, Structure of solids and liquids

## Abstract

Solid nitrogen exhibits a panoply of phenomena ranging from complex molecular crystalline configurations to polymerization and closing band gap at higher densities. Among the elemental molecular solids, nitrogen stands apart for having phases, which can only be stabilized following particular pressure-temperature pathways, indicative of metastability and kinetic barriers. Here, through the combination of Raman spectroscopy and dynamic compression techniques, we find that the appearance of the whole nitrogen phase diagram is determined by the *P-T* paths taken below 2 GPa. We reveal the existence of the path- and phase-dependent triple point between the $\beta$-$N_2$, $\delta _{loc}$-$N_2$ and $\gamma$- or $\epsilon$-$N_2$. We further show that the $\beta$-$N_2$ towards $\gamma$-$N_2$ path below the triple point, that evades $\delta$($\delta _{loc}$)-$N_2$, results in the formation of $\gamma$-$N_2$, which in turn becomes a dominant phase. We then demonstrate, that the $\beta$-$N_2$ through $\delta$($\delta _{loc}$)-$N_2$ above the triple point path leads to the formation of $\epsilon$-$N_2$ and the “well-established” phase diagram. An additional pathway, which by-passes the rotationally inhibited modifications $\delta$($\delta _{loc}$)-$N_2$, via rapid compression is found to produce $\gamma$-$N_2$ at higher temperatures. We argue that the pathway and phase sensitive triple point and the compression rate dependent phase formation challenge our understanding of this archetypal dense molecular solid.

## Introduction

The occurrence of polymorphism in the solid-state has long been a testing ground for modern calculations, encompassing our state-of-the-art understanding of the interplay of intermolecular interactions, volume minimization and finite temperature entropic effects^1–6^. Nitrogen, in likeness to other elemental molecular phase diagrams, such as hydrogen, oxygen and the halogens, demonstrates rich polymorphism^7–13^. This polymorphic abundance, particularly at low pressures and temperatures, is governed by intermolecular interactions, degree of rotational disorder permitted to the molecular units and their associated packing efficiency. Strikingly, in contrast to simple diatomic molecular systems, nitrogen exhibits numerous examples of molecular arrangements which can only be achieved through intricately crafted pressure-temperature pathways. In addition, these phases once stabilised can heavily influence nitrogen’s phase diagram, including the pressure-induced dissociation of its triple bond, the second strongest of any diatomic molecule, into polymeric states at higher densities. These effects can be attributed to a conjecture proposed over one century ago by the father of physical chemistry, Wilhelm Ostwald, that upon a phase transformation the system may not necessarily find the most stable state but rather the nearest (meta)stable one^14,15^. The observed (meta)stability in molecular nitrogen has resulted in one of the most complicated phase diagrams amongst the elements, which includes numerous molecular and non-molecular states, and consequently its equilibrium phase diagram remains challenging to constrain despite its rigorous investigation since the beginning of the $20{th}$ century.

**Figure 1:**
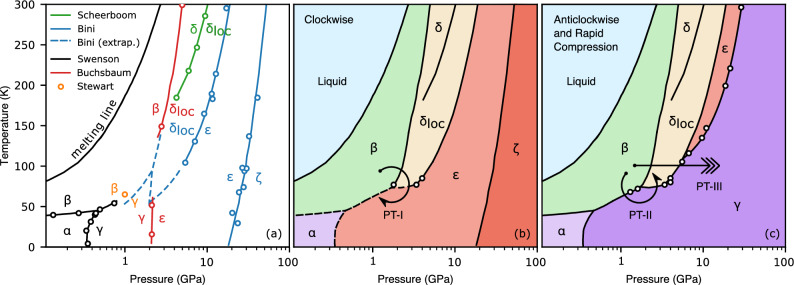
Transformation, kinetic and phase diagrams of nitrogen in a wide pressure and temperature range. (**a**) The currently accepted phase diagram of $N_2$. The solid lines and open symbols are from the Refs.^7–10,16–19^. The dashed lines are extrapolations from the Refs.^9,10^. (**b**) Proposed transformation and kinetic diagram of nitrogen. The clock-wise (above the triple point trough the rotationally inhibited phases) direction: formation of $\epsilon$-$N_2$. The $\gamma$-$N_2$ phase does not form and is replaced by $\epsilon$-$N_2$. (**c**) Proposed transformation and kinetic diagram of nitrogen. The counter-clockwise (below the triple point) direction: $\gamma$-$N_2$ forms replacing subsequent $\epsilon$-$N_2$, $\zeta$-$N_2$ and $\kappa$-$N_2$ phases found at higher pressures. The arrow shows the rapid compression *P-T* path also leading to formation of the $\gamma$ phase.

A novel molecular phase, known as $\lambda$-$N_2$, was recently discovered at low temperature^13^, Fig. S1. There are several interesting questions arising about the nature and properties of $\lambda$-$N_2$. Considering the vast amount of experimental work done on nitrogen it is intriguing why this phase was not seen before in the phase diagram^7–9^. Among all nitrogen phases known today, $\lambda$-$N_2$ is rather unique due to the intricate *P-T* path which has to be followed at low temperatures in order to form the phase. $\lambda$-$N_2$ was only found to be synthesized by compressing liquid nitrogen at 77 K and only recovered to room temperature above $\sim$32 GPa^13^. Interestingly, the Raman spectra of $\lambda$-$N_2$ appear to be almost identical to those of $\theta$-$N_2$^13,20^, however these phases are separated in the *P-T* space by almost 100 GPa and 1000 K at their formation, Fig. S1. The (meta)stability of the $\iota$, $\theta$ and $\lambda$ modifications is also striking: even though $\lambda$ forms at low temperature it can be recovered at 300 K; once recovered it occupies the whole phase space of $\epsilon$, $\zeta$ and $\kappa$^12^, the same way the $\iota$ and $\theta$ do once quenched from the high temperature. Also, if heated at around 70 GPa $\lambda$-$N_2$ would transform to the $\theta$-$N_2$ phase^13^.

Here, we report that the phase claimed as $\lambda$-$N_2$ is in fact the previously known low temperature-pressure phase - $\gamma$-$N_2$. However, we demonstrate that $\gamma$-$N_2$ is of fundamental importance in shaping the subsequent nitrogen phase diagram to extreme pressures and temperatures. We observe that even subtle changes in trajectory along *P-T* pathways between 0 and 2 GPa can result in two distinct entirely different nitrogen phase diagrams. A triple point at 66 K (± 5 K) and 1.8 GPa (± 0.2 GPa) is identified and attributed as the mechanism responsible, it is found to lie at the intersection of three different classes of molecular phases characterised by the degree of their rotational ordering: (1) complete disorder - $\beta$-$N_2$, (2) partially ordered - $\delta$($\delta _{loc}$)-$N_2$ and (3) completely ordered $\gamma$-$N_2$ or $\epsilon$-$N_2$. We demonstrate that this triple point is sensitive to the *P-T*-path undertaken and how it is navigated determines what is the resulting fully ordered molecular phase, either $\gamma$-$N_2$ or $\epsilon$-$N_2$. By utilising dynamic compression techniques, we also show that the formation of $\gamma$-$N_2$ is considerably more complicated than just the compression of nitrogen at low temperature and that the kinetics plays an important role in formation of the $\gamma$ phase. We synthesised $\gamma$-$N_2$ at temperatures up to 100 K through rapid compression (0.4 TPa/s), bypassing $\delta$($\delta _{loc}$)-$N_2$ and consequently inhibiting the formation of $\epsilon$-$N_2$. Through extrapolation we speculate that $\gamma$-$N_2$ could also be formed at room temperature provided a sufficient compression rate. This work brings new insights into the nature of solid nitrogen at high pressures and together with calculated enthalpies for respective molecular phases suggest that the nitrogen equilibrium phase diagram could be remarkably simple and therefore more compatible with its diatomic molecular family.

## $\gamma$-$N_2$ and its place on the phase diagram

The existence of the $\gamma$-$N_{2}$ phase was inferred almost 70 years ago from discontinuities in nitrogen’s compressibility at low temperatures^17^ and its boundary between its neighboring phases, $\beta$-$N_{2}$ and $\alpha$-$N_{2}$, was constrained. Over a decade later x-ray diffraction experiments suggested that $\gamma$-$N_2$ has a tetragonal structure with $P4_2/mnm$ symmetry^21,22^. Subsequent Raman scattering experiments were carried out from 15-300 K and up to 52 GPa^16^ in order to map the molecular phase diagram. In these pioneering experiments the optical data were collected through isobaric scans, iterating cooling and warming cycles at different target pressures which were defined at room temperature. In these measurements they report that $\gamma$-$N_2$ appears in only a limited pressure and temperature range between 0.4 and 2 GPa and up to 60 K, Fig. 1a. Crucially they report the “quite surprising” existence of a new phase line, observing the co-existence of two phases with substantially different molecular arrangements, $\gamma$-$N_{2}$, which is a simple structure exhibiting only a few low frequency Raman modes, and the much more complex $\epsilon$-$N_{2}$. More recent Raman and IR spectroscopy measurements have also re-investigated the phase diagram of nitrogen in detail through isobaric scans^7–10^, Fig. 1a, which to date comprise our modern understanding of the low temperature nitrogen phase diagram. It is important to note that the phase boundaries shown with the dashed blue lines in Fig. 1a were extrapolated^9,10^ down to lower pressures and temperatures (see the area of the diagram around 2 GPa and 50 K in Fig. 1a) and likely drawn in a way to avoid a thermodynamically forbidden quadruple point between $\beta$-$N_{2}$, $\delta _{loc}$-$N_{2}$, $\epsilon$-$N_{2}$ and $\gamma$-$N_{2}$.

**Figure 2:**
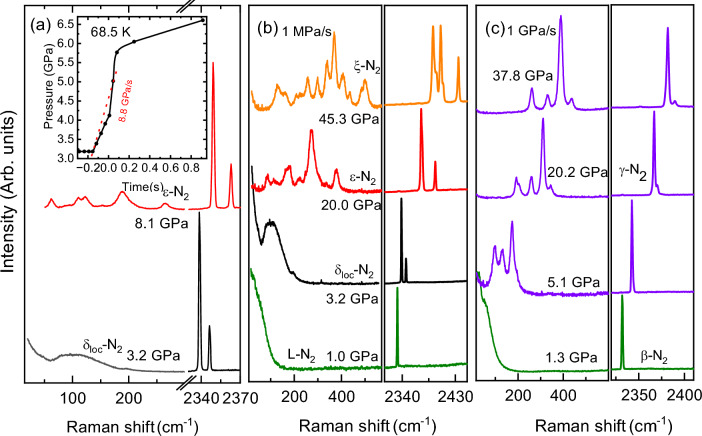
Formation of $\gamma$-$N_2$ versus $\delta _{loc}(\epsilon )$-$N_2$ as a function of the starting states and compression rate. (**a**) Transformation of $\delta _{loc}$-$N_2$ to $\epsilon$-$N_2$ at 8.8 GPa/s and 68 K. (**b**) Transformation of $\beta$-$N_2$ to $\delta _{loc}$-$N_2$ at 1 MPa/s, and then to $\epsilon$-$N_2$ and $\zeta$-$N_2$ at 77 K. (**c**) Transformation of $\beta$-$N_2$ to $\gamma$-$N_2$ at 1 GPa/s at 68 K.

In our studies, we have followed both the isothermal and isobaric *P-T* paths, observing in the latter case the same placement and sequence of the phases as described in the literature^7,9,10,16^, however the existence of a second triple point was never observed. It is important to note that in the isobaric experiments the $\gamma$-$N_{2}$ phase can only be reached upon cooling directly from the $\beta$-$N_{2}$ phase, but if the *P-T* path crosses the phase space of $\delta$($\delta _{loc}$)-$N_{2}$ then only $\epsilon$-$N_{2}$ is formed (see Figs. 1 and 2b). Therefore, the formation of $\gamma$-$N_{2}$ or $\epsilon$-$N_{2}$ depends on the initial phase being $\beta$ or $\delta$.

Nevertheless, the picture changes if one follows the isothermal *P-T* path, Figs. 1c and 2. Firstly, we consider the temperatures below the temperature of the $\beta$-$\delta$-$\epsilon$/$\gamma$-$N_{2}$ triple point ( 66 ± 5 K and 1.8 ± 0.2 GPa). If compression starts anywhere in the stability field of $\gamma$-$N_2$, $\le$ 60 K and $\le$2 GPa, then we observe the smooth evolution of the Raman spectra of the vibrational and lattice modes, continuously increasing in frequency with pressure, Fig. S2. Four lattice modes have the same origin point, but become fully resolved by around 5 GPa. At these *P-T* conditions the spectrum of $\gamma$-$N_{2}$ is indistinguishable from that of the $\lambda$-$N_{2}$ phase, *c.f.* also with Ref.^13^. Theoretical structural searches at 0 K^3^ found energetically very competitive $P2_{1}/c$-4 configuration, which later was confirmed as a possible solution for the $\lambda$-$N_2$ structure^13^. On the other hand, the original x-ray study proposed $P4_2/mnm$-4 as the structural candidate for the $\gamma$-$N_{2}$ phase^21^. In Fig. S3a,b we show the $P4_2/mnm$-4 and $P2_{1}/c$-4 structural models and note that they have the same body-centred based lattice (if the the objects on the body centres are molecules, rather than atoms) except $P2_{1}/c$-4 does not have a $90^{\circ }$ angle. In both cases they represent a simple configuration of 2 molecules. However, according to our calculations the more symmetric $P4_2/mnm$-4 is less energetically stable than $P2_{1}/c$-4 and is predicted to have only two lattice modes, while we observe four (Figs. S2 and S3), therefore it could be ruled out as a structural model for $\gamma$-$N_2$. If compression starts at slightly higher temperatures in the $\beta$-$N_{2}$ phase (just below the triple point) then we observe the transformation to $\gamma$-$N_2$, when the phase line is crossed and continuous evolution of the $\gamma$-$N_2$ phase with pressure as described above.

## Synthesis of $\gamma$-$N_2$ by fast compression

We have noticed very interesting and unusual behaviour when compressing nitrogen above the triple point e.g. T$\ge$66 K starting in the $\beta$-$N_2$ phase. The expectation would be that upon compression the $\beta$ phase first transforms to $\delta$ followed by $\epsilon$, Fig. 1a. However, in some cases at temperatures slightly higher than that of the triple point, we have observed the formation of $\gamma$-$N_2$ upon compression, while in other cases $\epsilon$-$N_2$ was formed. The phase transformations at temperatures above 66 K seem to be driven by the kinetic effects and compression rate starts to play the role in the phase formation mechanism. It appears that the rate at which the pressure is increased defines if $\gamma$-$N_2$ would form or not instead of the traditionally expected epsilon phase.

**Figure 3:**
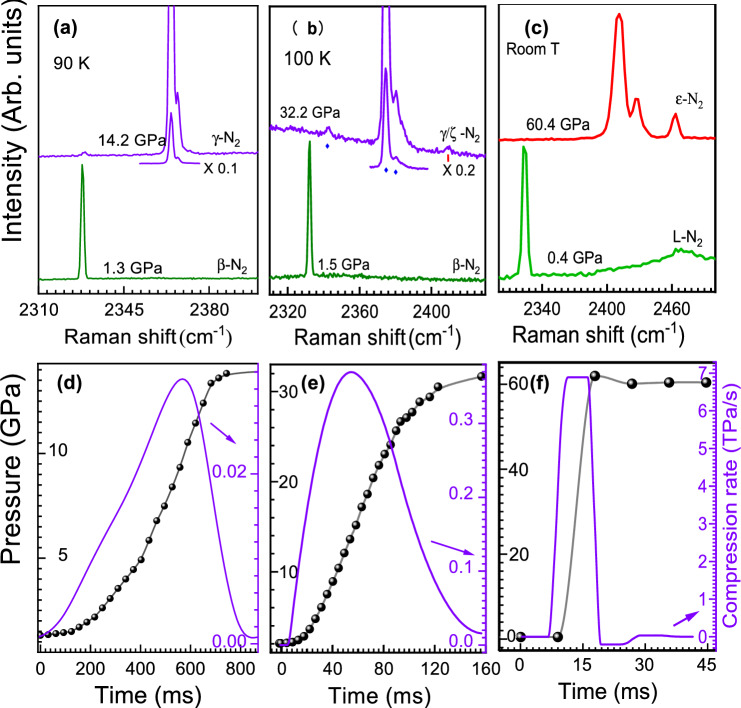
Fast compression of nitrogen at different temperatures. Upper panels: Raman spectra of nitrogen before and after compression. Lower panels: the corresponding compression rates (violet curve, y-axis on the right in TPa/s units) and pressures (black curve with solid circles) versus time. (**a**) and (**d**) T=90 K, starting pressure 1.3 GPa ($\beta$ phase), the peak compression rate 30 GPa/s, final pressure 14 GPa, $\gamma$-$N_2$ formed. (**b**) and (**e**) T=100 K, starting pressure 1.5 GPa ($\beta$-$N_2$ phase), the peak compression rate 370 GPa/s, final pressure 32 GPa, $\gamma$-$N_2$ and small addition of $\zeta$-$N_2$ formed. (**c**) and (**d**) T=300 K, starting pressure 0.4 GPa (L-$N_2$), the peak compression rate 7 TPa/s, final pressure 60 GPa, $\epsilon$-$N_2$ formed.

The $\delta _{loc}$-$N_2$ phase appears above the triple point, and it is wedged between $\beta$ and $\epsilon$, see Fig. 1. If compression starts in the $\beta$ phase and is slow (e.g. several MPa/s) then the stability range of $\delta _{loc}$ is crossed slowly - the time scale of tens of seconds or minutes. In this case nitrogen will have sufficient time to transform into $\delta _{loc}$ first and then into $\epsilon$, Fig. 3a,b (see also Figs. S4 and S5). Conversely, if pressure is increased with the rate of several GPa/s, then the pressure stability range of $\delta _{loc}$ can be traversed relatively quickly (less than a second) and $\gamma$-$N_2$ is formed, Fig. 2c. The $\gamma$ phase appears as soon as the $\delta _{loc}$-$\epsilon$ phase line is crossed and $\gamma$ is formed instead of $\epsilon$, Fig. 2c. In order to produce $\gamma$-$N_2$ at even higher temperatures one still needs to start the pressure increase from either liquid- (L-) or $\beta$-$N_2$, rapidly by-passing the $\delta _{loc}$ phases and ending in the stability field of $\epsilon$, see Figs. 1 and S1. Figures 2 and 3 demonstrate that if compression starts (or ends) in the region of $\delta$($\delta _{loc}$) or the compression rate is too low for a given temperature then we observe the “standard” phases such as $\epsilon$. If the starting state of nitrogen is the $\delta _{loc}$ or $\epsilon$ phase, $\gamma$-$N_2$ cannot be synthesised at any temperature or compression rate.

**Figure 4:**
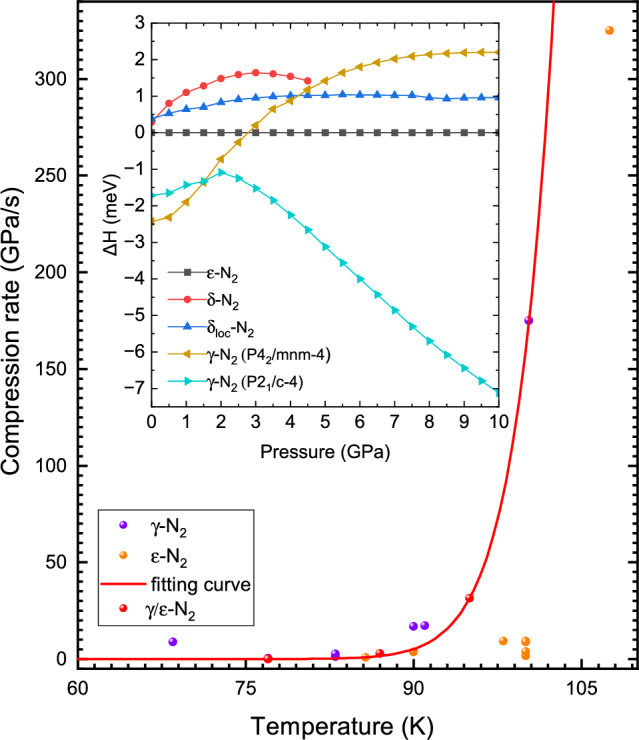
The minimal compression rate required to synthesize the $\gamma$-$N_2$ versus temperature and the enthalpies of the nitrogen phases. The measured compression rate points are shown as filled circles, with different colours signifying the final phases. The red solid curve is an exponential fit (Arrenius equation). The compression rate was obtained by averaging out the compression rate during the compression in the $\delta$($\delta _{loc}$)-$N_2$ phase space. Insert: enthalpies of the low pressure-temperature nitrogen phases at 0 K from 0 to 10 GPa.

Note that the region of $\delta$($\delta _{loc}$) which needs to be by-passed becomes broader with temperature, reaching pressure of 32 GPa at 300 K, Figs. 1 and S1. To quickly pressurise $N_2$, we have used a fast compression technique (described in detail in Ref.^23^ and in the "Methods" section), which allows us to increase pressure in diamond anvil cell with the rates up to 10 TPa/s ($10^4$ GPa/s) at 300 K. Figure 3 shows the Raman spectra collected at 100 and 300 K, see also Figs. S4 and S5. At 100 K, a very high temperature previously believed to preclude the formation of $\gamma$, the minimum compression rate needed to produce it is on the order of 400 GPa/s (0.4 TPa/sec). In Fig. 4 we plot the compression rates which led to the synthesis of $\gamma$-$N_2$ at different temperatures. Up to $\sim$66 K the speed of pressure increase essentially does not play any role and the formation of the phase is governed only by whether the compression started at pressures below the pressure of the triple point (2 GPa, $\gamma$ or $\beta$) or above ($\delta$ or $\epsilon$). But as soon as temperature is above 74 K, particularly above the temperature of the liquid nitrogen, the compression rate starts to rise exponentially. It is natural to apply so called Arrhenius equation^24–29^

$$\upsilon =C\exp \left( -\frac{\Delta E_g}{k_BT}\right)$$

to the exponential dependency observed in our experiments, Fig. 4. Even though it is usually used when *P* is constant, one can adapt it to describe phase transformation when pressure is changing (see Refs.^27–29^). In the equation above $\Delta E_g$ is an activation energy, *C* is a constant related to the material, $k_B$ is the Boltzman constant and $\upsilon$ is a phase growth rate at a given temperature^27–29^ . By fitting two parameters we obtain the activation energy $\Delta E_g=25.6 \mathrm{kJ/mol}$ and *C*=3.8$\times$ $10^{15}$ GPa/s. In our experiments we have tried to synthesise $\lambda$ at room temperature achieving the rates of around 7 TPa/s (Fig. 4), which is one of the highest rates ever reported^23^ but the resulting phase was still $\epsilon$-$N_2$. Extrapolating the Arrhenius equation with $\Delta E_g$ and *C* for nitrogen to 300 K one can obtain the compression rate of about $10^6$ TPa/s. This is the minimal compression rate needed to produce $\gamma$ at room temperature, which is clearly beyond the current capabilities of diamond anvil cell.

In this and our previous studies^13^ we have investigated the stability of $\gamma$($\lambda$) on decompression. We have found that on down-stroke there is a phase line, beyond which (at lower pressures) $\gamma$($\lambda$) does not exist. It is interesting to note that this line lies entirely in the stability field of $\epsilon$ (shown as dashed line in Fig. 1b) loosely tracking another phase line between $\epsilon$ and $\delta _{loc}$ all the way to around 100 K where it joins $\delta _{loc}$-$\epsilon$ boundary. It appears that this line plays an important role in the $\gamma$ phase formation at high temperatures because one needs to by-pass not only $\delta _{loc}$ but also the narrow region within the $\epsilon$ phase space. For example, in order to synthesize $\gamma$ at 300 K one would need to “jump” to above 32 GPa, the pressure at which $\gamma$ back-transforms to $\epsilon$ on decompression at 300 K^13^.

## Nitrogen phase diagram as a function of *P-T* direction, rate of compression and Ostwald’s rule

It is informative to analyse the response of nitrogen and resulting phase appearance to the fast compression. During the phase transition formation of nucleation sites and grain growth rate depend on the activation energy barriers^14,15,27–31^, which in turn are a function of the *P-T* path and compression rate. The compression rate can slow down or even completely prevent the nucleation. But if pressure is changing fast the system might not have enough time for the nucleation sites to form and grow into another configuration. Therefore, compression time, the characteristic transition time $\tau$ and the free energies of the phases involved are the determining factors for the phase transformation to take place^14,15,27–31^. In inset to Fig. 4 we plot the enthalpies of the concerned phases relative to $\epsilon$-$N_2$ calculated at 0 K. We have used the $P2_{1}/\mathrm{c-4}$ structure originally proposed for the structure of $\lambda$-$N_2$ in our calculations^3,13^. There are several important points to note from the graph: (1) the structure originally proposed for the $\gamma$ phase $P4_2/mnm-4$ is stable only to about 2 GPa, while we observe $\gamma$-$N_2$ all the way to 130 GPa; (2) only $\beta$- and $P2_{1}/\mathrm{c-4}$ have lower energies than $\epsilon$ in the pressure range studied (2 to 10 GPa); (3) $\delta$ and $\delta _{loc}$ have always higher energies than $\epsilon$; (4) $P2_{1}/\mathrm{c-4}$ is essentially identical energetically and is the most stable above 1.5 GPa with lower enthalpy at higher pressure.

The phase relationship and transformations described above suggests a specific free-energy landscape which has two distinct and very close in energy minima corresponding to $\epsilon$ and $\gamma$ phases, who are in competition in the same *P-T* space, being separated by $\delta$($\delta _{loc}$). Their formation is determined by the direction of travel around the $\beta$-$\delta$-$\gamma$($\epsilon$) triple point. In fact, the appearance of one of the phases which makes the triple point depends on the direction in which the point is being traversed around. If the “standard” compression rate is used e.g. on the level of a MPa per second and the *P-T* path lies through $\delta$-$N_2$ (clockwise) then $\epsilon$ is formed and the spheres- and disk-like phases become dominant in the phase diagram. There are no any pathways to convert it to $\gamma$ because it is kinetically hindered. On other hand if the triple point is travelled around in the counter-clockwise manner then the ordered molecular $\gamma$ phase wins and occupies around 90$\%$ of the phase diagram between ambient pressure and up to $\sim$150 GPa in 0 K to $\sim$ 300 K.

The picture becomes very interesting if one follows clockwise path and crosses the stability field of $\delta$($\delta _{loc}$), which is less stable than either $\gamma$ or $\epsilon$. In this case the formation of a given phase would be determined by the competition between how fast $\delta$($\delta _{loc}$) transforms to $\epsilon$ and $\beta$ transforms to $\gamma$. Since $\gamma$ is a low temperature phase the faster compression rates are required at higher temperatures to jump over the $\delta$($\delta _{loc}$) stability field in order to prevent the grain nucleation and growth of $\gamma$ phase. In some of our experiments when $\gamma$ was formed we observed an additional vibrational peak belonging to $\zeta$ (see Fig. 3b), which can happen for two different reasons. If the jump over $\delta$($\delta _{loc}$) lands very close to the line beyond which $\gamma$ does not exist (Fig.1c), small parts of the sample back transform to $\epsilon$ and then at higher pressure to $\zeta$. Or if the compression rate is very close or slightly below the critical one shown in Fig. 4, the nucleation growth can happen resulting in the activation behavior for the $\epsilon$ phase.

To the best of the authors knowledge the interplay between the low pressure - low temperature phases of nitrogen described here is rather unusual and very rare if known at all. The remotely similar picture is realised in highly condensed sodium at 118 GPa^32^, where multiple phases could be accessed by a slight change of pressure or temperature. Here, two solid phases separated by less than 2 meV (at 4 GPa) can be “switched” either by the direction of the *P-T* path or more interestingly “mechanically” by the speed of compression, which is required to bypass the phases with slightly higher (only by 1 meV) free energy. This concept of “phase switching” in accordance with the Ostwald’s rule that the preferred phase formed would have the lowest kinetic barrier to the parent phase. Our study demonstrates that the $\gamma$ phase, which has been known for several decades and previously thought to occupy only a minuscule part of the phase diagram is in fact probably the prominent feature of condensed molecular nitrogen.

## Methods

### DAC preparation and low temperature

We have conducted more than 40 independent high-pressure experiments at temperature varying from 10 to 300 K and pressures up to 60 GPa. Both long piston-cylinder (Mao-Bell type) and symmetric diamond anvil cells were used equipped with diamonds with culet diameters between 200 and 500 $\mu$m. Stainless steel (T304) sheet and rhenium foils of 200-250 $\mu$m initial thickness served as gaskets. After gasket indentation from the diamonds, sample chambers were laser-machined and high purity nitrogen gas (99.999$\%$) was loaded at 2.0 kbars together with an annealed ruby sphere as a pressure marker. Pressures were determined using the ruby pressure scale^33^ with a corresponding low temperature correction^34^. Sample pressures were either manipulated through either screw rotation, high pressure He-gas in a membrane or piezoelectric actuation, the latter is discussed in the following section).

The low temperature experiments were carried out using either a wet cryostat from cryoindustries or a custom dry cryostat. Two Photec/Lakeshore Si-diodes thermometers were used to measure the temperature as well as provide feedback to the Photec Tmon8 temperature controller. One diode was attached to the bore/coldfinger of the respective cryostat, whilst the second was mechanically fixed to the DAC body in close proximity to the sample chamber. To reduce the systematic error due to temperature gradients, temperatures were allowed to stabilise for a period of $\sim$20 minutes prior to data collection.

### Fast compression experiments

Slow compression, up to 10 GPa/sec, was found to the typical compression rate achieved in screw-driven experiments at 300 K. To reach much higher compression rates in a wide temperature range (10-300 K), diamond anvil cells were driven and controlled by either stepper motors, pneumatic membranes or piezoelectric actuators. Each dynamic control compression system has their respective advantages at different temperature conditions^23^. For instance, although piezoelectric actuators provide the fastest compression rate at room temperature, stepper motors and pneumatic membranes were used at low termpatures as they are easier to integrate with the cryostat.

The compression rates were determined by analyzing *in situ* time-resolved ruby fluorescence spectra during the compression process. The ruby fluorescence was captured by our optical set-up described below in the section on the Raman spectroscopy, capable of $\sim$1 ms time resolution. The pressures and compression rates in dynamic compression experiments were extracted from the ruby fluorescence data and their associated timestamps stored in the files metadata. Characteristic compression curves from these experiments are represented in Figs. 3, 4, S4 and S5.

### Raman spectroscopy

Raman scattering experiments were performed with a custom confocal Raman system, which has been described in previous work^35–37^. This system consisted of a Spectra Pro 750-mm monochromator equipped with a Charge-coupled Device (CCD) detector (Princeton Instruments). The Raman spectra were collected with a high-resolution 1800 g/mm grating. Largely a 532 nm DPSS laser from Laser Quantum was used as an excitation source for the Raman emission, however in some experiments and additional 660 nm DPSS laser from Laser Quantum was incorporated.

### DFT calculations

DFT calculations were performed with castep^38^ using on-the-fly generated norm-conserving pseudopotentials. We used a 1000 eV plane-wave cut-off and regular $\textbf{k}$-point grids formed from points spaced 0.03$\times 2\pi$ $\text{\AA }^{-1}$ apart. Raman frequencies and intensities were obtained using density functional perturbation theory (DFPT) implemented in castep^39^.

### Supplementary Information

Supplementary Information.

## Data Availability

The data that support the findings of this study are available from the corresponding author upon request.
