# Supplementary material for: Remarkable stability of γ-N2 and its prevalence in the nitrogen phase diagram
Source: Sci Rep. 2024 Jul 16;14:16394. doi: 10.1038/s41598-024-66493-0 (PMC11252275; doi:10.1038/s41598-024-66493-0)
Supplement: Supplementary file 1 — Supplementary Information. [file 41598_2024_66493_MOESM1_ESM.pdf]

Supplementary material for: Remarkable Stability of γ-N_2 and its Prevalence in the Nitrogen Phase Diagram

Jinwei Yan,[1,2,3,4] Philip Dalladay-Simpson,[2*] Lewis J. Conway[5,6], Federico Gorelli,[2]
Chris Pickard[5,6], Xiaodi Liu,[1*] and Eugene Gregoryanz[1,2,3]

[1]*Key Laboratory of Materials Physics,*
Institute of Solid State Physics, HFIPS,
Chinese Academy of Sciences, Hefei 230031, China

[2]*Center for High Pressure Science and Technology Advanced Research, Shanghai, China*

[2]*Centre for Science at Extreme Conditions and School of Physics an Astronomy,*
University of Edinburgh, Edinburgh, U.K.

[4]*University of Science and Technology of China, Hefei, China*

[5]*Department of Materials Science and Metallurgy, University of Cambridge,*
27 Charles Babbage Road, Cambridge CB30FS, UK and

[6]*Advanced Institute for Materials Research,*
Tohoku University, Sendai, 980-8577, Japan[*]

Transformation and kinetic phase diagram of molecular N_2 in a wide P-T range

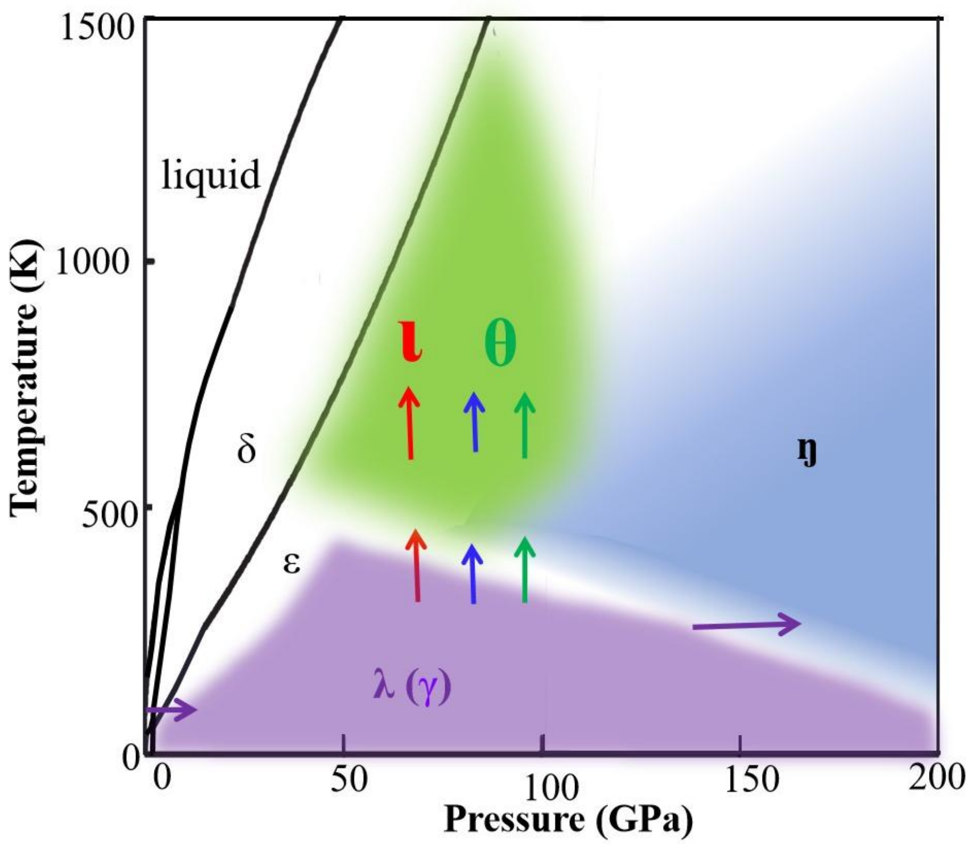

FIG. S1. Schematic transformation and phase diagram of nitrogen in a wide pressure and temperature range. $\epsilon(\zeta)$ or $\gamma(\lambda)$-N_2 transform upon heating to the molecular ι or θ-N_2 or non-molecular η-N or cg-N (not shown). Adopted from [1–3].

Raman spectra of γ-N_2 compared to theoretical calculations

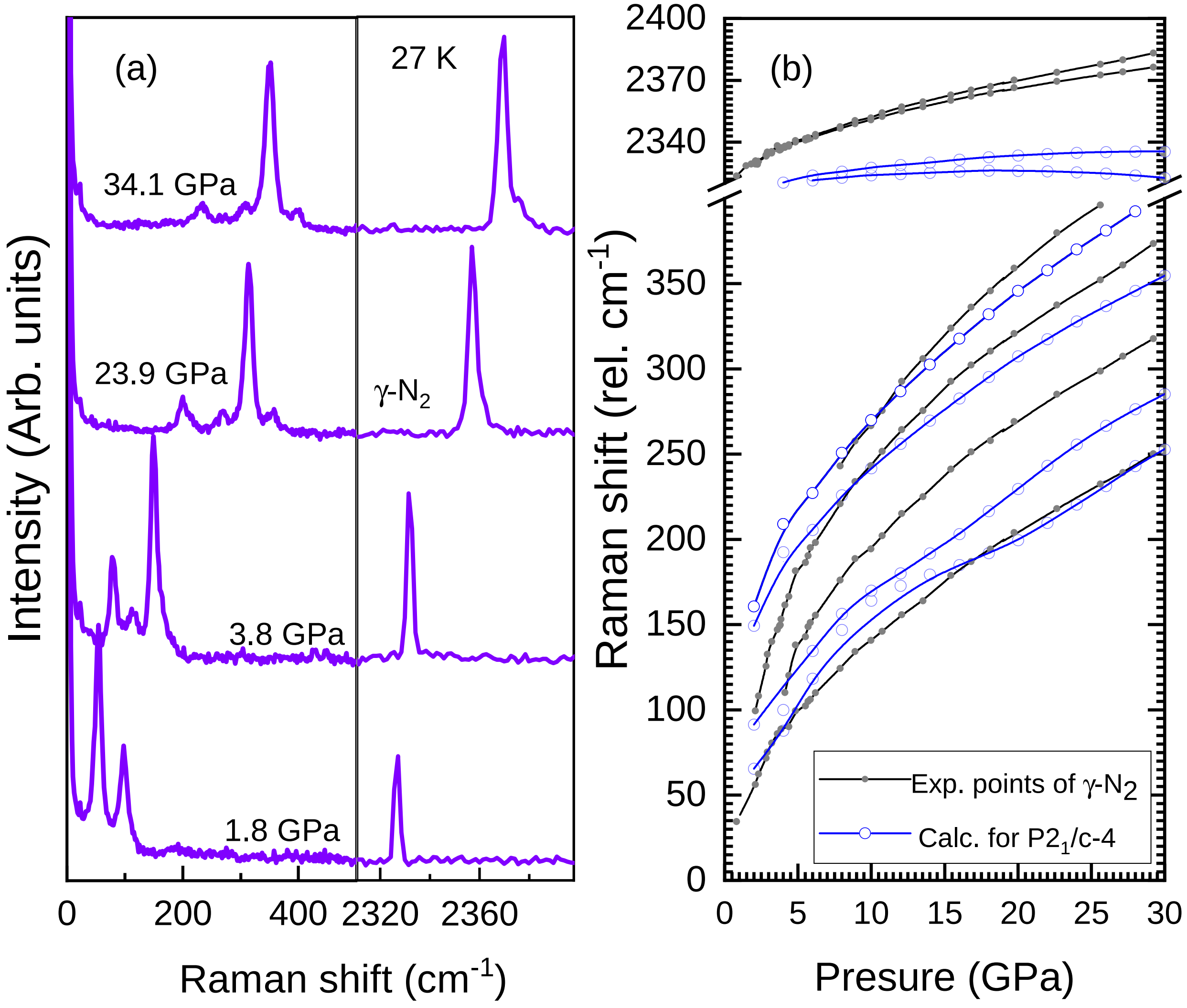

FIG. S2. The evolution of γ-N_2 during isothermal compression. (a) Raman spectra of γ-N_2 evolved with pressure at 27 K (below the triple point). (b) Frequencies of lattice and vibrational Raman modes as a function of pressure from experimental measurements of γ-N_2 and theoretical calculation for $P2_1/c$-4 model. Note that the frequencies versus pressure curves are identical to those of λ-N_2 [4]

Structure of γ-N_2

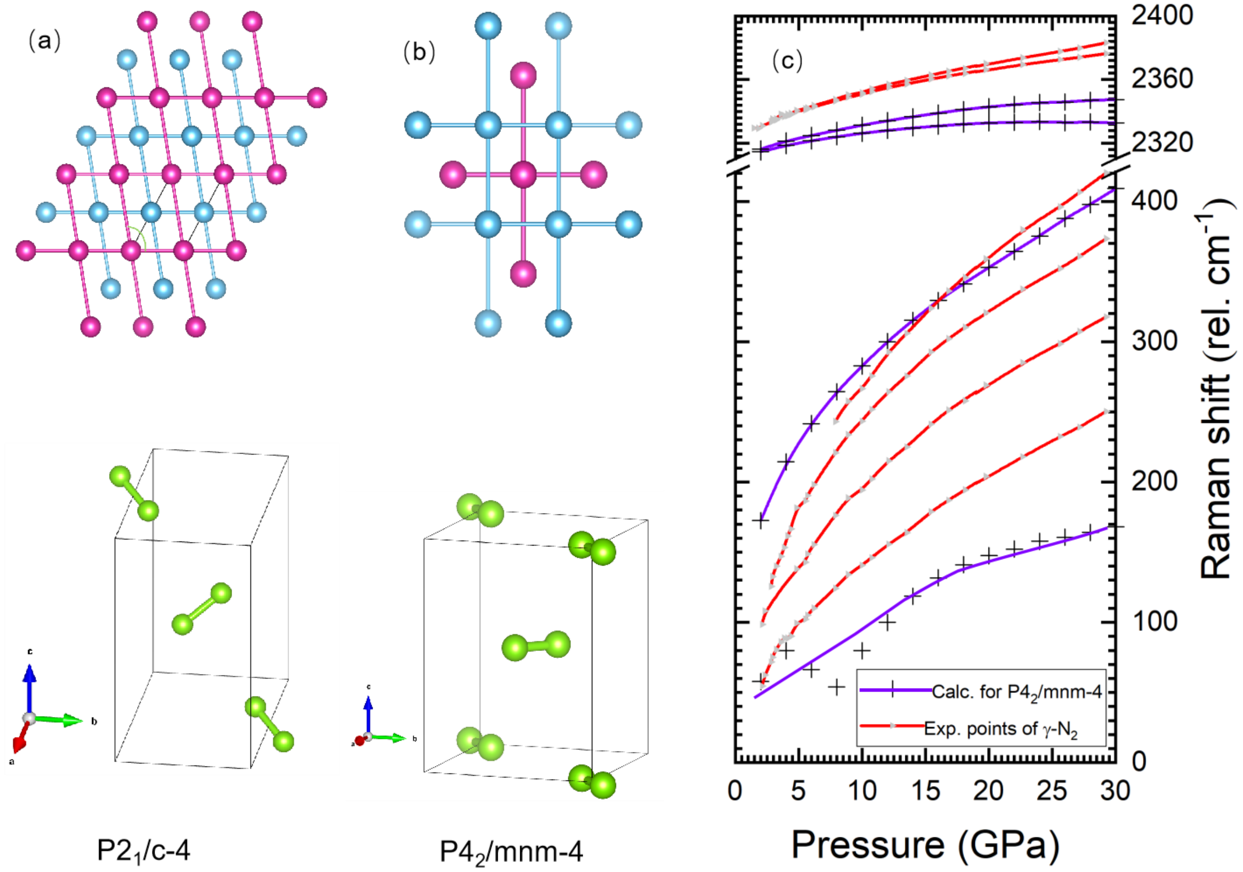

FIG. S3. The unit cell of P2$_1$/c-4 (a) and P4$_2$/mnm-4 (b). (c) Red curves: experimentally measured frequencies of the lattice modes of γ-N_2 versus pressure. Violet curves: calculated frequencies for the P4$_2$/mnm-4 structure.

Representative Raman spectra of N_2 collected along various *P*-*T* paths

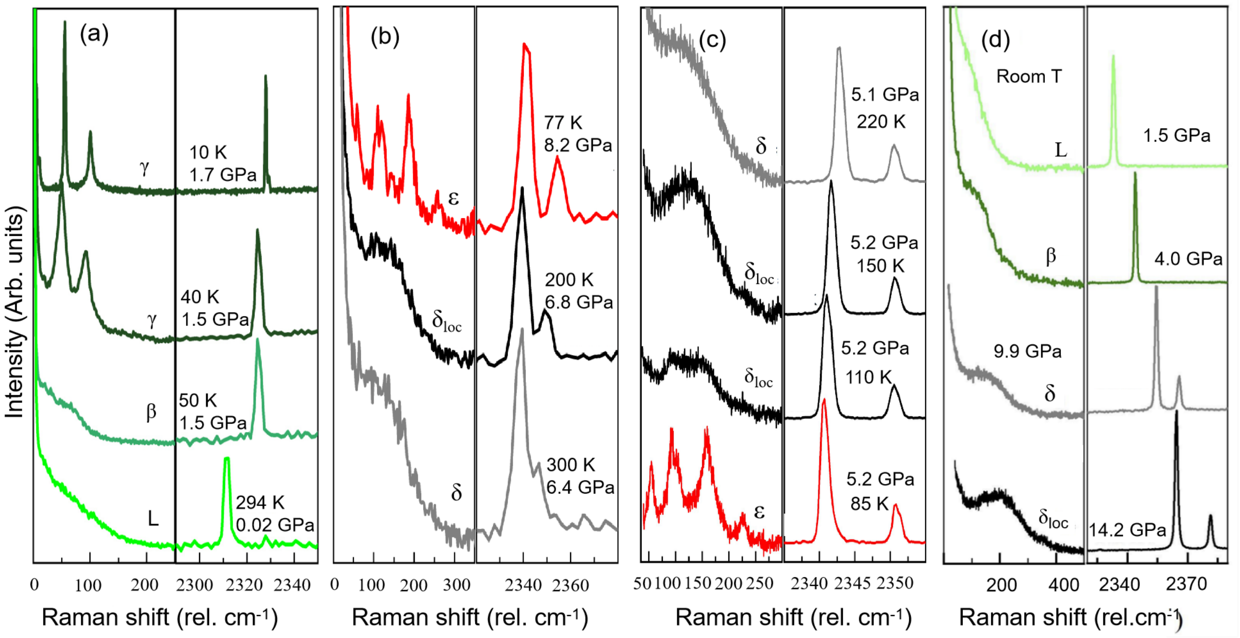

FIG. S4. Some of the *P*-*T* paths taken in this work, used to construct phase diagram in Fig. 1. The corresponding *P*-*T* conditions are marked for each spectrum.

Representative Raman spectra of N_2 collected along various P-T paths with different compression rates

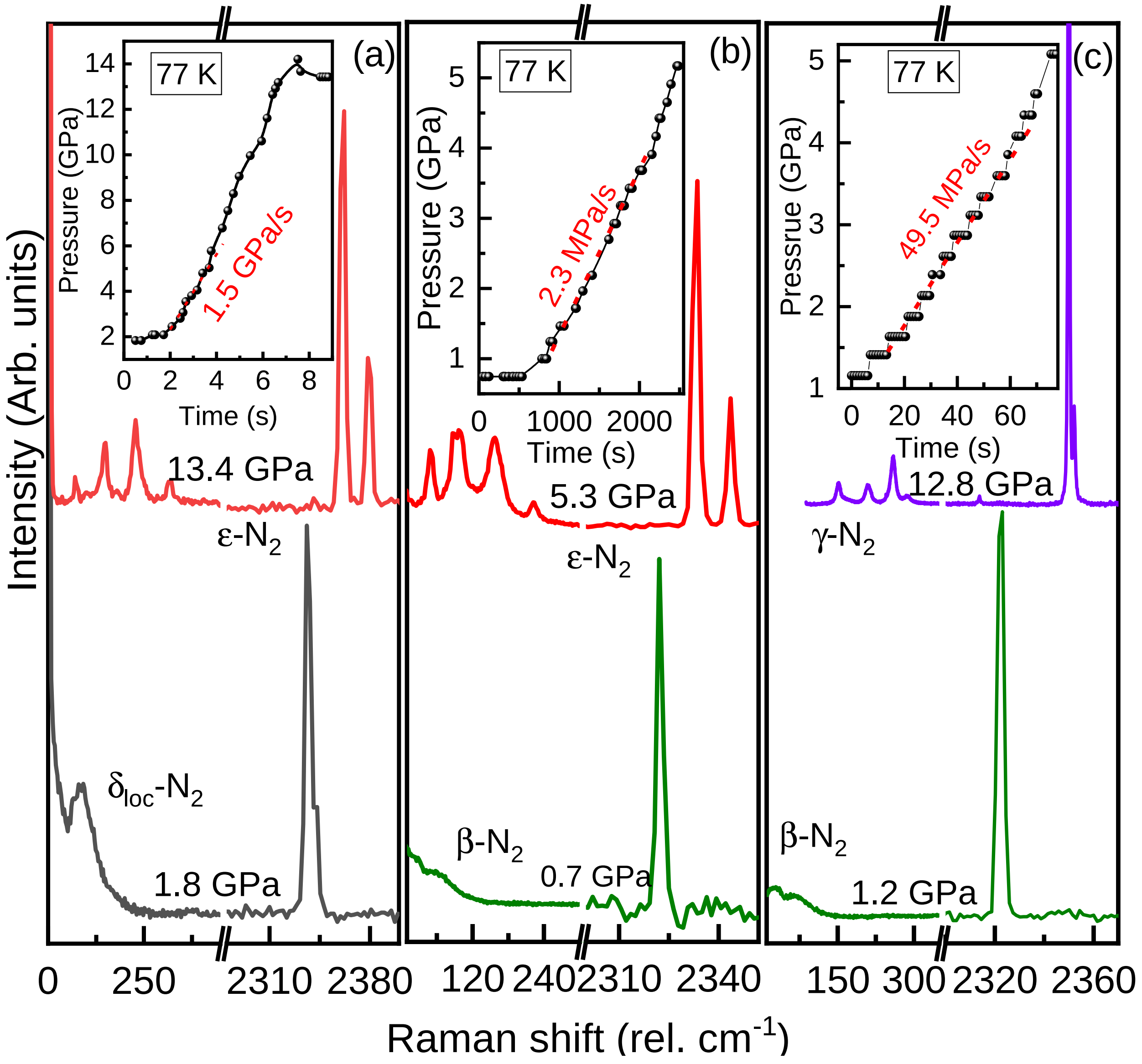

FIG. S5. Representative Raman spectra of the corresponding phase before and after compression. The P-T conditions are marked for each spectrum. Insets: pressure change versus time.

Representative Raman spectra of N_2 collected along various P-T paths with different compression rates

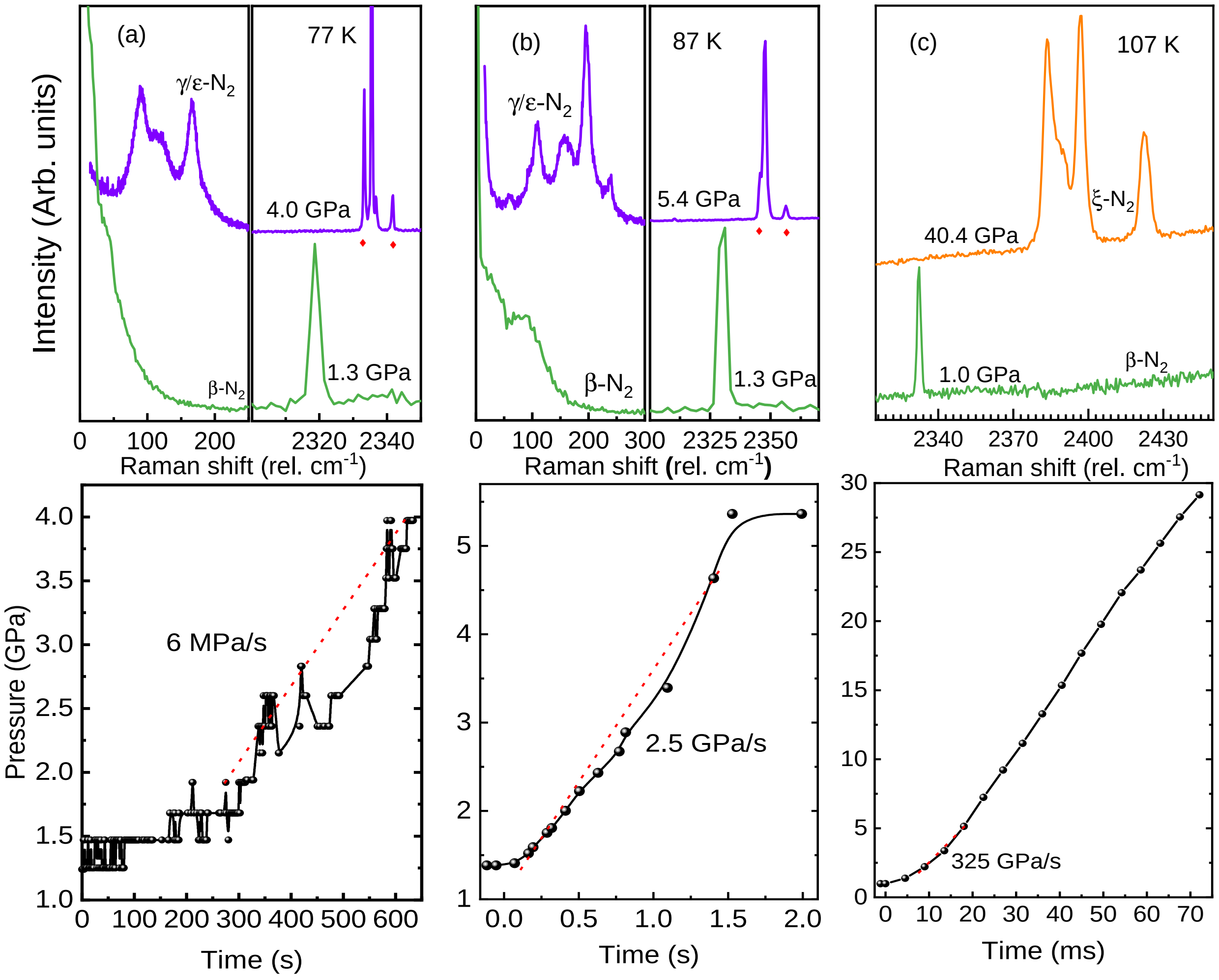

FIG. S6. Upper panels: Representative Raman spectra of the corresponding phases before and after compression. Lower panels: The change of pressure versus time for the spectra in the upper panel. The P-T conditions are marked for each each spectrum.

*

[1] E. Gregoryanz, A. F. Goncharov, R. J. Hemley, H.-k. Mao, M. Somayazulu, and G. Shen, Phys. Rev. B **66**, 224108 (2002).

[2] M. Frost, R. T. Howie, P. Dalladay-Simpson, A. F. Goncharov, and E. Gregoryanz, Phys. Rev. B **93**, 024113 (2016).

[3] R. Turnbull, M. Hanfland, J. Binns, M. Martinez-Canales, M. Frost, M. Marques, R. T. Howie, and E. Gregoryanz, Nat. Commun. **9** (2018).

[4] R. Bini, L. Ulivi, J. Kreutz, and H. J. Jodl, J. Chem. Phys. **112**, 8522 (2000).
